# Note on Pancreatin

**Published:** 1873-12

**Authors:** Richard V. Mattison


NOTE ON PANCREATIN—*By Richard V. Mattison.*—An elixir being suggested for the exhibition of this in an elegant form, the following formula was devised, and we think will be found very agreeable:

| | |
|---|---|
| Pancreas, . . . . . . . . | No. vj. |
| Acid Hydrochlor, . . . . . . . | f℥iv. |
| Glycerin, . . . . . . . . | q. s. |
| Aqua . . . . . . . . . | Cong. iss. |

Macerate the dissected pancreas for three days in the mixture of water and acid with Oiiss of glycerin added; then separate the liquid, strain and add f℥iiss oil of orange and a sufficient quantity of glycerin to make the liquid measure Cong. iiss; this is then filtered until perfectly transparent. The result is a sweet acidulous elixir, one fluidrachm of which will easily emulsify half a fluid-ounce of cod liver oil—a valuable addition to the number of preparations combining efficiency with pharmaceutical elegance.

